# THE INFLUENCE OF RECESSION HARDSHIPS ON THE PSYCHOSOCIAL WELL-BEING OF SANDWICHED AND FILIAL CAREGIVERS

**DOI:** 10.1093/geroni/igz038.2282

**Published:** 2019-11-08

**Authors:** Barbara Hodgdon, Jen D Wong

**Affiliations:** The Ohio State University, Columbus, Ohio, United States

## Abstract

The 2007-2009 U.S. Great Recession impacted the lives of many families, and it has been documented that multigenerational households in the U.S. increased by 10% during this period. Given the vulnerability of providing care to multiple generations, there is a need to examine the influence of recession hardships on sandwiched caregiving and psychosocial well-being in the context of more normative caregiving (e.g., filial caregiving). Informed by the life course perspective, this study assessed the impacts of types of family caregiving (sandwiched and filial caregivers) on psychosocial well-being (e.g., affect, environmental mastery, and social actualization) and the moderating role of recession hardships (e.g., job loss, foreclosure). Sandwiched and filial caregivers (N=127; Mage=53; SD=11.02) from the Refresher Cohort of the Midlife in the United States (MIDUS) Survey provide information on demographics, recession hardships, family caregiving, and well-being. Results from regression analyses showed that sandwiched caregivers exhibited lower levels of positive affect and environmental mastery than filial caregivers. Moderation analyses showed that filial caregivers with lower recession hardships exhibited lower positive affect and social actualization when compared to sandwiched caregivers with lower recession hardships. Filial caregivers with lower recession hardships exhibited lower positive affect and social actualization than sandwiched and filial caregivers with greater recession hardships. These results illustrate the complexity of family caregiving in that providing care to multiple generations does not necessarily translate to lower levels of well-being. Study findings have the potential to inform programs that may promote sandwiched caregivers’ well-being and support filial caregivers navigating financial disruptions.

